# Absolute and Relative Socioeconomic Health Inequalities across Age Groups

**DOI:** 10.1371/journal.pone.0145947

**Published:** 2015-12-30

**Authors:** Sander K. R. van Zon, Ute Bültmann, Carlos F. Mendes de Leon, Sijmen A. Reijneveld

**Affiliations:** 1 Department of Health Sciences, Community & Occupational Medicine, University Medical Center Groningen, University of Groningen, Antonius Deusinglaan 1, 9700 AD, Groningen, The Netherlands; 2 Department of Epidemiology, University of Michigan School of Public Health, 1415 Washington Heights, Ann Arbor, Michigan, The United States of America; Hunter College, UNITED STATES

## Abstract

**Background:**

The magnitude of socioeconomic health inequalities differs across age groups. It is less clear whether socioeconomic health inequalities differ across age groups by other factors that are known to affect the relation between socioeconomic position and health, like the indicator of socioeconomic position, the health outcome, gender, and as to whether socioeconomic health inequalities are measured in absolute or in relative terms. The aim is to investigate whether absolute and relative socioeconomic health inequalities differ across age groups by indicator of socioeconomic position, health outcome and gender.

**Methods:**

The study sample was derived from the baseline measurement of the LifeLines Cohort Study and consisted of 95,432 participants. Socioeconomic position was measured as educational level and household income. Physical and mental health were measured with the RAND-36. Age concerned eleven 5-years age groups. Absolute inequalities were examined by comparing means. Relative inequalities were examined by comparing Gini-coefficients. Analyses were performed for both health outcomes by both educational level and household income. Analyses were performed for all age groups, and stratified by gender.

**Results:**

Absolute and relative socioeconomic health inequalities differed across age groups by indicator of socioeconomic position, health outcome, and gender. Absolute inequalities were most pronounced for mental health by household income. They were larger in younger than older age groups. Relative inequalities were most pronounced for physical health by educational level. Gini-coefficients were largest in young age groups and smallest in older age groups.

**Conclusions:**

Absolute and relative socioeconomic health inequalities differed cross-sectionally across age groups by indicator of socioeconomic position, health outcome and gender. Researchers should critically consider the implications of choosing a specific age group, in addition to the indicator of socioeconomic position and health outcome, as findings on socioeconomic health inequalities may differ between them.

## Introduction

Reducing health inequalities related to socioeconomic position (SEP) is a major challenge globally [1–3]. In general, people with a low SEP are at an increased risk for negative health outcomes [1]. Socioeconomic health inequalities have been found for various outcomes like morbidity [4,5], mortality [6], and measures of physical and mental health [7,8].

SEP refers to resources and prestige linked to the social class of individuals, expressed in indicators like education and income [9]. Education might influence health through health-related knowledge, literacy, skills, occupational opportunities and thereby income, while income might influence health through the ability to purchase healthy food, live in better housing and obtain medical care [9–11]. Indicators of SEP affect health throughout the life course, operating at different levels and through different pathways [9–11]. It is well known that indicators of SEP are not necessarily interchangeable as their associations with health outcomes have been shown to differ [12–16].

Age is another factor that affects the magnitude of socioeconomic health inequalities. Previous studies have shown that socioeconomic health inequalities in industrialized countries tend to be small in young adulthood, become larger in midlife, and then decrease again at older ages [7,17–21]. Differences in socioeconomic health inequalities across age groups may be due to aging itself but they may also be caused by differences between cohorts [17,22]. Literature suggests that age is more important in relation to health at older ages while SEP may be more important to health at younger ages [23]. Differences in the distribution of educational level across cohorts may affect findings on socioeconomic health inequalities as a certain educational level may have a different meaning, and thereby relation to health, across cohorts [23–25]. In general, the educational level of younger age groups is higher than the educational level of older age groups. For instance in the Netherlands in 2010, 83% of the 25–34 years old versus 61% of the 55–64 years old completed at least an upper secondary education [26], indicating that cohort effects may exist. Income might be more related to age itself. Income tends to increase from adolescence to adulthood, being highest during midlife, and then typically decreases after retirement [27]. Individuals with a low income in early adulthood might be at the start of their career because they have many years of schooling, implying that income might increase in later life, whereas this is unlikely for individuals with a low income at midlife [27,28]. A low income may thus also have a different meaning, and thereby relation to health, across age groups.

To date, it is unclear whether socioeconomic health inequalities across age groups differ by other factors that are known to affect the relation between SEP and health, like the indicator of SEP, the health outcome [12–16], gender [13], and as to whether socioeconomic health inequalities are measured in absolute terms (i.e. the difference in rates or means between socioeconomic groups for a certain outcome) or relative terms (i.e. the ratio of rates for a certain outcome across socioeconomic groups) [29–32]. Therefore, the aim of this study is to investigate whether absolute and relative socioeconomic health inequalities differ across age groups by indicator of SEP (education and income), health outcome (physical and mental health), and gender in a large-scale cross-sectional sample of Dutch individuals.

## Methods

### Study design and sample

The study sample was derived from the LifeLines Cohort Study [33,34]. LifeLines is a multi-disciplinary prospective population-based cohort study examining in a unique three-generation design the health and health-related behaviors of 167,729 persons living in the North East region of the Netherlands. It employs a broad range of investigative procedures in assessing the biomedical, socio-demographic, behavioral, physical and psychological factors which contribute to the health and disease of the general population, with a special focus on multi-morbidity and complex genetics.

Details on recruitment and data collection procedures have been described elsewhere [34]. In short, individuals were invited to participate by their general practitioner or through family members, and there was an option to self-register. Participants filled out questionnaires and visited one of the LifeLines research centers for a physical examination. The present study uses data from participants who visited the LifeLines research centers between November 2006 and March 2013 for the baseline measurement. LifeLines was conducted according to the guidelines in the Declaration of Helsinki and all procedures involving human subjects were approved by the Medical Ethics Committee of the University Medical Center Groningen. Written informed consent was obtained from all participants.

### Measures and procedures

The RAND-36 was used to measure physical and mental health [35]; this has been shown to have good reliability and validity [36]. The RAND-36 contains 36 items measuring eight health domains: physical functioning, role limitations caused by physical health problems, role limitations caused by emotional problems, social functioning, emotional well-being, energy/fatigue, pain, and general health perceptions [35]. Each domain is scored separately from 0 to 100 with lower scores indicating poorer health. The domain scores were standardized by linear z-score transformation to have a mean of 50 and a standard deviation (SD) of 10 in the US general population [37,38]. Two summary component scores, the physical component score (PCS) and the mental component score (MCS), were constructed from these eight domains using recommended scoring algorithms, with all domains contributing to both summary scores [38]. The PCS primarily reflects measures of physical functioning, pain, and role limitations caused by physical health problems while the MCS primarily reflects measures of emotional well-being and role limitations caused by emotional problems. General health perceptions, energy/fatigue, and social functioning are reflected in both component scores [38]. The PCS and MCS were also dichotomized into poor health (PCS/MCS <50) and good health (PCS/MCS ≥50).

SEP was defined by educational level and household income. Educational level was measured according to the Dutch classification for education with a single-item question regarding the highest educational level achieved and had eight response categories. Educational level was categorized into primary education (no education, primary education), lower secondary education (lower or preparatory vocational education, lower general secondary education), higher secondary education (intermediate vocational education or apprenticeship, higher general senior secondary education or pre-university secondary education), and tertiary education (higher vocational education, university) [39]. Household income was measured using the following question: “How much is your net income per month? If you share the costs with someone, then add the net income of your partner(s) to your income” with eight response categories. Household income was categorized into a household income <€1000 per month, a household income between €1000 - €2000 per month, a household income between €2000 - €3000 per month, and a household income >€3000 per month.

Age was calculated based on the date of the first clinical visit. In 2009, the average age for leaving the educational system was 21 years in Europe (range: 17–24 years) and 22.5 years in the Netherlands (range: 19.5–25 years) [28]. We included participants aged ≥25 years as those were assumed to have their own SEP. Age was categorized into eleven 5-years groups.

### Statistical analyses

First, baseline characteristics were examined using descriptive statistics, i.e. percentages and means ± SD. Second, absolute socioeconomic inequalities in physical and mental health were examined by comparing mean PCSs and MCSs using educational level and household income as separate indicators of SEP. Analyses were performed for all age groups, and stratified by gender. Third, relative socioeconomic inequalities in physical and mental health were examined by calculating Gini-coefficients. The Gini-coefficient is a measure of relative inequality based on the Lorenz curve [40–42]. Traditionally, the Lorenz curve shows the cumulative percentage of income by the cumulative percentage of the population, but the curve can also be used for health outcomes [40–42]. If health is equally distributed among individuals, the Lorenz curve is a diagonal line (i.e. perfect equality). The more the Lorenz curve deviates from the diagonal, the larger the degree of inequality [40–42]. See Porta [40] or Regidor [41] for a visual representation of the Lorenz curve. The Gini-coefficient is measured on a scale from 0 to 1, where 0 represents complete equality (i.e. health is equally distributed among individuals or groups) and 1 complete inequality (i.e. all health of the population is concentrated within a single person or group) [40–42]. The Gini-coefficient can be calculated by dividing the area between the diagonal and the Lorenz curve by the total area under the diagonal [40]. Another approach to calculate the Gini-coefficient, applied in this paper, is by using categorical variables. Instead of using the Lorenz curve, the area under the Receiver Operating Characteristic (ROC) curve (AUC) is used to calculate the Gini-coefficient [41,43]. With this approach, the Gini-coefficient is based on the proportion of people with a certain health state (e.g. poor health) across socioeconomic groups. If the proportion of people with poor health is equal across socioeconomic groups, the AUC is zero. The more the proportion of people with poor health varies across socioeconomic groups, the larger the AUC, and thereby the larger the Gini-coefficient will be. The AUC values were obtained from the logistic regression procedure in SAS (Proc Logistic), using the dichotomized PCS and MCS as outcome and educational level and household income as predictor. Then, the AUC was transformed into a Gini-coefficient by the following equation: Gini-coefficient = 2 * AUC– 1 [43]. These analyses were performed for all age groups, and stratified by gender. Analyses were performed using SPSS (version 22; IBM Corp., Armonk, NY) and SAS software (version 9.3; SAS Institute Inc., Cary, NC, USA).

## Results

### Baseline characteristics

**Fig 1:**
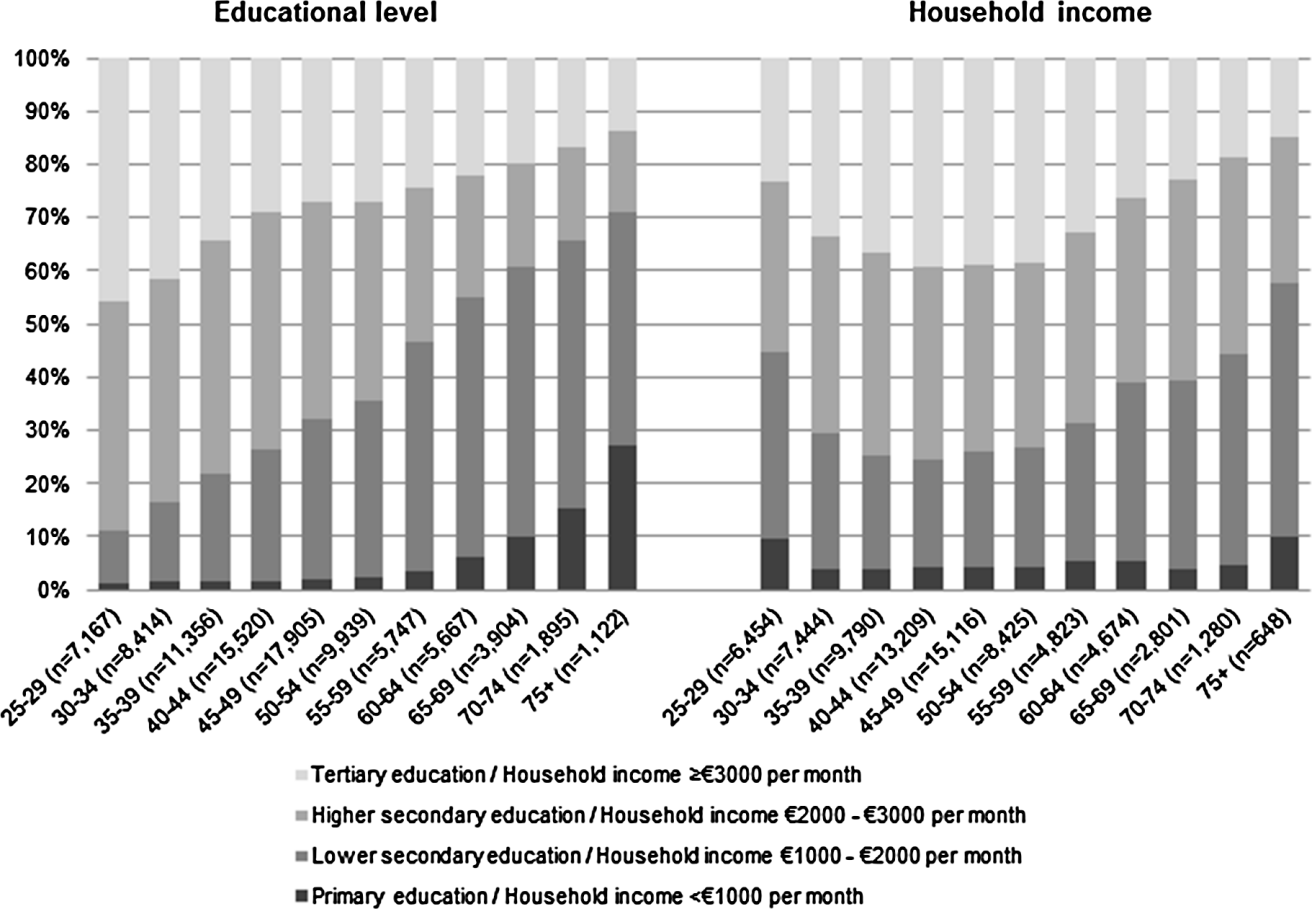
Educational level and household income by age group.

A total of 95,432 participants were included, of whom 58.7% were female (Table 1). The mean age of the study sample was 44.7 years (SD: 12.6). Mean PCS and MCS were 51.3 (7.3) and 52.5 (8.4), respectively. Younger age groups were higher educated than older age groups, while the distribution of household income was inverse U-shaped (Fig 1).

**Table 1:** Baseline characteristics of the study sample.

	N	Mean (SD) or %
Age	95,432	44.7 (12.6)
Gender, F	95,432	58.7
Educational level	93,134	
Primary education	2,859	3.1
Lower secondary education	26,226	28.2
Higher secondary education	36,752	39.5
Tertiary education	27,297	29.3
Household income per month	78,571	
<€1000	5,991	7.6
€1000 - €2000	19,635	25.0
€2000 - €3000	27,109	34.5
≥€3000	25,836	32.9
Physical component score	92,920	51.3 (7.3)
Poor physical health	26,303	28.3
Mental component score	92,920	52.5 (8.4)
Poor mental health	22,837	24.6

### Absolute inequalities across age groups by indicator of SEP, health outcome and gender

**Fig 2:**
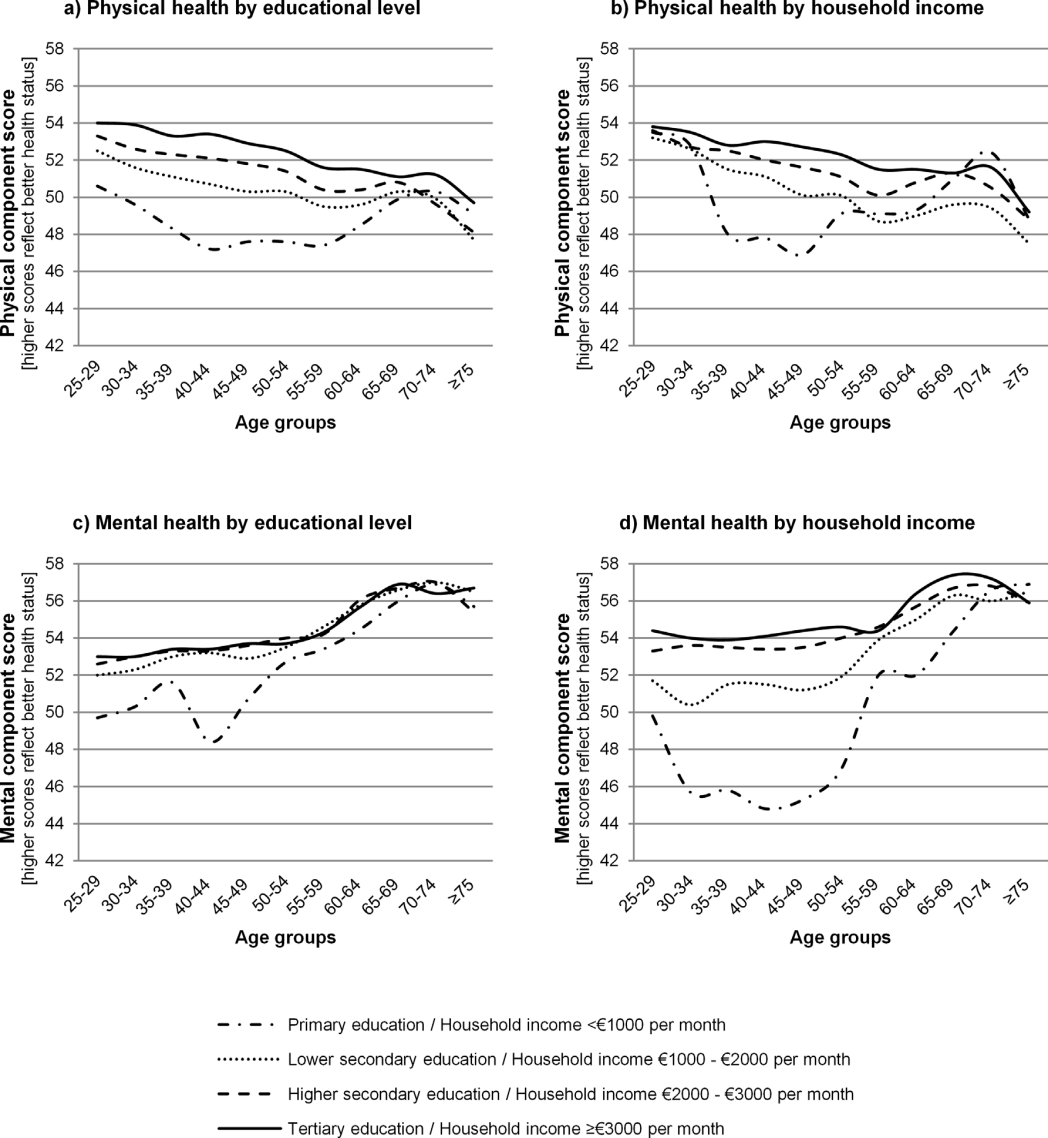
Absolute inequalities in physical and mental health across age groups by indicator of socioeconomic position for males.

**Fig 3:**
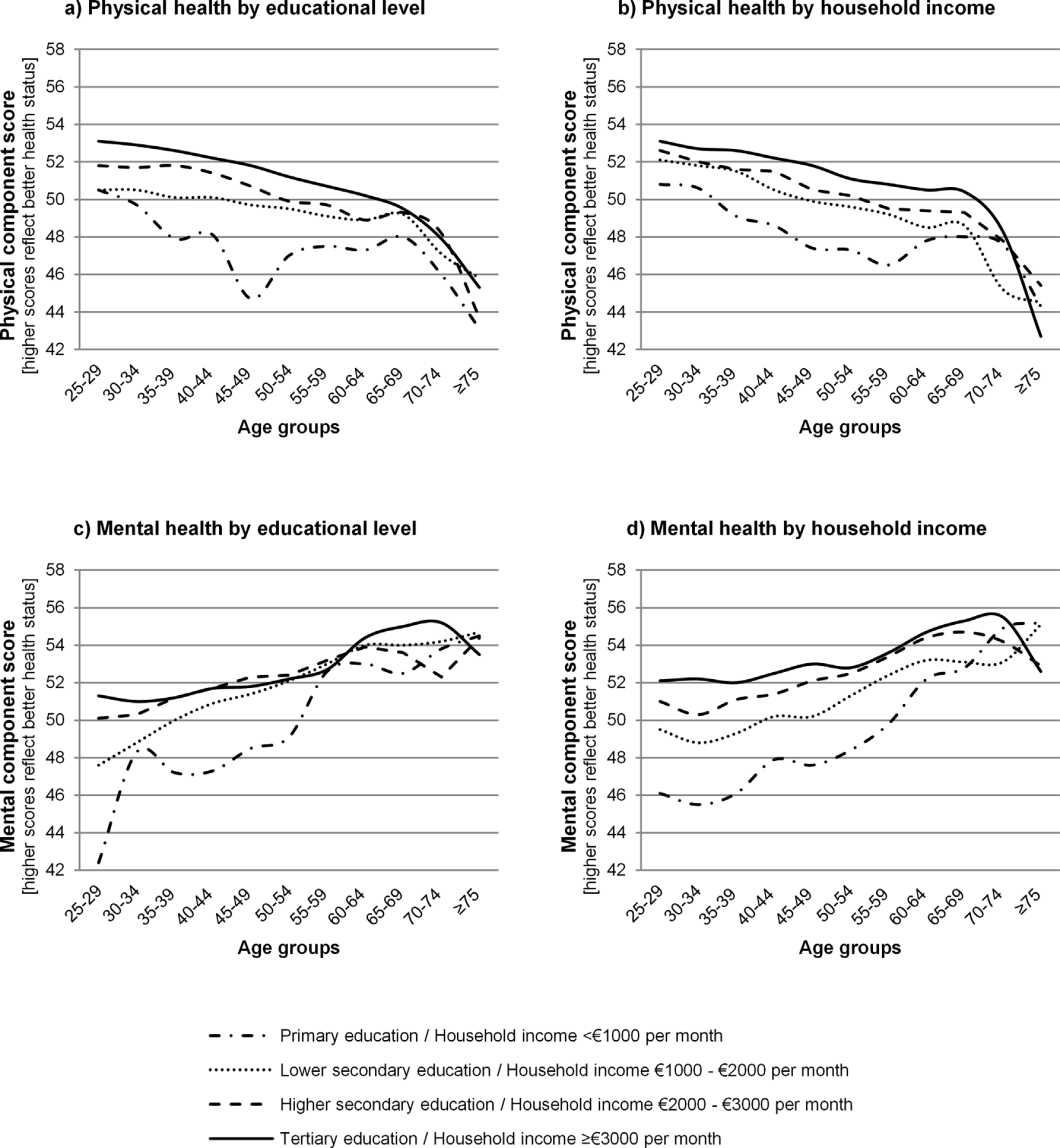
Absolute inequalities in physical and mental health across age groups by indicator of socioeconomic position for females.

Absolute socioeconomic health inequalities tended to differ across age groups by indicator of SEP, health outcome and gender (Figs 2 and 3 and S1A–S1J Table). For example, in 40–44 year old males, the absolute difference in PCS between males with a tertiary education and males with a primary education was 6.2 points while this absolute difference was 1.2 in 65–69 year old males (Fig 2A). This means that absolute socioeconomic inequalities in physical health were larger in 40–44 year old males than in 65–69 year old males.

Socioeconomic health inequalities between age groups were most pronounced for mental health by household income. Inequalities were larger in younger compared to older age groups (Fig 2D for males; Fig 3D for females). Absolute physical health inequalities were smaller in young and old age groups compared to middle age groups for both indicators of SEP, especially for males (Fig 2A and 2B for males; Fig 3A and 3B for females).

Absolute inequalities in physical health were fairly similar for educational level (Fig 2A for males; Fig 3A for females) and household income (Fig 2B for males; Fig 3B for females), although absolute inequalities tended to be smaller by household income than by educational level in females, but not in males. Absolute inequalities in mental health were smaller by educational level than by household income, both for males (Fig 2C and 2D) and females (Fig 3C and 3D).

Absolute inequalities were different by health outcome, regardless of the indicator of SEP. Physical health inequalities were larger than mental health inequalities by educational level, especially for males (Fig 2A;c for males; Fig 3A;c for females). But physical health inequalities were smaller than mental health inequalities by household income (Fig 2B;d for males; Fig 3B;d for females).

There were some differences between males and females. For example, a U-shape was found for the MCS by household income between the age group 25–29 years and the age group 55–59 years in males but not females.

### Relative inequalities across age groups by indicator of SEP, health outcome and gender

**Fig 4:**
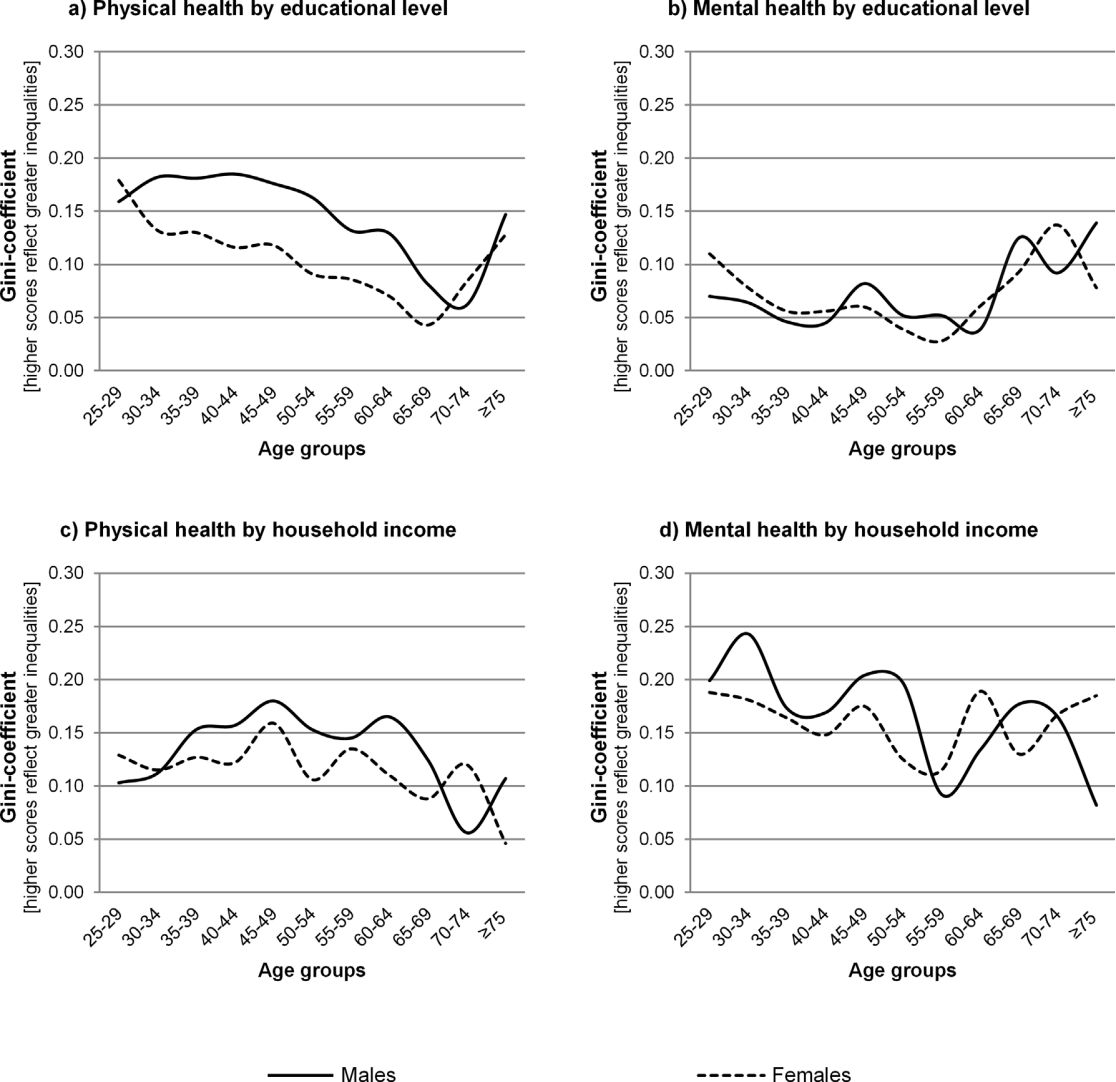
Relative inequalities in physical and mental health across age groups by indicator of socioeconomic position and gender.

Relative socioeconomic health inequalities differed across age groups by indicator of SEP, health outcome and gender (Fig 4 and S1A–S1J Table). For example, in 40–44 year old males, the Gini-coefficient for physical health, with educational level as indicator of SEP, was 0.18 while the Gini-coefficient was 0.06 in 70–74 year old males (Fig 3A). This means that poor physical health (PCS <50) was more unequally distributed among socioeconomic groups in 40–44 year old males than in 70–74 year old males. Physical health inequalities by educational level tended to be smaller in older compared to younger age groups, although inequalities were somewhat larger in those aged ≥70 compared to those aged 65–69 (Fig 4A). Inequalities were larger for males than females. Mental health inequalities by educational level were smaller in those aged 30–64 years (range Gini-coefficients: males: 0.04–0.08; females: 0.03–0.08) than in those aged ≥65 years (range Gini-coefficients: males: 0.09–0.14; females: 0.08–0.14) (Fig 4B).

Physical health inequalities by household income were smaller in younger and older age groups, compared to the age groups in between (35–64), for males but not for females (Fig 4C). Inequalities for females were, despite some fluctuations, similar across age groups. Mental health inequalities by household income fluctuated across age groups without a clear pattern (Fig 4D). These fluctuations seemed larger for males than females.

Relative inequalities in physical health differed between educational level and household income (Fig 4A;4C). Physical health inequalities by educational level were smaller in older compared to younger age groups in both males and females. For males, physical health inequalities by household income were largest in those aged 35–64. Inequalities were fairly stable across age groups for females. Mental health inequalities were also different between educational level and household income (Fig 4B;4D). For educational level, mental health inequalities were overall small, while mental health inequalities by household income were overall large, both for males and females.

Relative inequalities differed by health outcome, regardless the indicator of SEP. Relative inequalities were larger for physical health than for mental health by educational level (Fig 4A and 4B). Moreover, physical health inequalities were smaller in older age groups (except for those aged ≥70), whereas mental health inequalities were larger in older age groups. Regarding household income, physical health inequalities tended to be smaller than mental health inequalities (Fig 4C and 4D). Moreover, for males, physical health inequalities were larger in those aged 35–64 compared with those younger and older, whereas no clear pattern was found for females, nor for mental health inequalities.

## Discussion

The aim of this large-scale cross-sectional study was to investigate whether absolute and relative socioeconomic health inequalities differed across age groups by indicator of SEP, health outcome and gender. Both absolute and relative inequalities differed across age groups by indicator of SEP, health outcome, and gender.

Reijneveld et al [25] previously showed that age modifies the relationship between educational level and SRH on the multiplicative scale. Studies specifically investigating socioeconomic health inequalities with ageing showed that health inequalities, measured as SRH, functional status, physical health or mental health, are small in young adulthood and then become larger in midlife [7,17–20,44]. Although findings on socioeconomic health inequalities in old age are less clear, most studies show narrowing socioeconomic health inequalities in old age [21]. We found a similar cross-sectional pattern for absolute inequalities in physical health for males and females, in mental health by household income for males, and in mental health by educational level for females. In this study, the increase in absolute health inequalities in midlife seems to emerge from reductions of health scores in the lowest educational and income groups. Although these groups are considerably smaller than the other groups, they encompass a substantial proportion of the population. Mental health inequalities were smaller in older compared to younger age groups by educational level in males and by household income in females. For relative inequalities, we only found a similar pattern for physical health by household income for men. This suggests that previously described patterns [7,17–20,44], might differ by indicator of SEP, health outcome, gender, and by measuring inequalities in absolute or relative terms.

We found a difference in relative mental health inequalities by educational level between those aged 30–64 years and those aged ≥65 years. Inequalities tended to be larger in those aged ≥65 years. Previous studies found that retirement results in better SRH [45] and in a decrease of physical and mental fatigue [46]. However, mental health improvements with ageing and after retirement, may be restricted to those with a high SEP [8,47]. This could possibly explain the larger inequality in mental health in participants aged ≥65 years compared to 30–64 years in this study; those with a higher educational level gain more from retirement. No difference in inequality in mental health was found for household income in participants aged ≥65 years compared to younger age groups. A possible explanation could be the comprehensive pension system in the Netherlands, although this is speculation.

Our finding that absolute and relative socioeconomic health inequalities may differ between indicator of SEP and between health outcome is in line with previous research [12–16]. For example, in a study by Davey Smith et al [14], occupational social class was a better discriminator of socioeconomic differences in mortality than education. Education was however most strongly associated with death from non-CVD and non-cancer causes [14]. Geyer et al [16] demonstrated that education was the strongest predictor for diabetes while income was the strongest predictor for all-cause mortality. Macintyre et al [13] showed that socioeconomic variations in health depended on the measurement of SEP, health outcome, and gender by comparing associations between seven indicators of SEP and five health outcomes. In older individuals, educational level or occupational social class, in combination with a deprivation indicator were shown to be the best indicators to measure socioeconomic health inequalities [15].

This study showed that socioeconomic health inequalities also differ if measuring them in absolute or in relative terms. For example, mental health inequalities by household income became clearly smaller with increasing age group if measured in absolute terms whereas there was no clear pattern if measured in relative terms. Moser et al [30] previously showed that the magnitude of socioeconomic health inequalities differs upon whether a rate ratio or rate difference is used. Barros et al [31] and Harper et al [32] also stress that conclusions on socioeconomic health inequalities might differ depending on whether an absolute or relative measure is chosen, which is in line with our findings.

The strengths of this study are the large sample size and the use of a reliable and valid measure of physical and mental health. The large sample size allowed analyses for 11 age groups, four categories of SEP, and stratified by gender. To our knowledge, no previous study was able to do this. This study also has some limitations. First, the use of the Gini-coefficient in research into socioeconomic health inequalities has been criticized because the Gini-coefficient alone does not reflect whether an individual with the worst health belongs to the lowest or highest SEP [41]. However, mean scores of physical and mental health and the prevalence of poor physical and mental health were consistently in favor of those with a higher SEP. Thus, the Gini-coefficient in this study does reflect the socioeconomic inequalities as expected; health outcomes are worse for those with a low SEP compared to those with a high SEP. Second, we could not determine whether differences in socioeconomic health inequalities across age groups were due to age or cohort effects [48]. In cross-sectional data, differences between age groups may be due to aging itself but they may also be caused by differences between cohorts [17,22]. Disentangling age and cohort effects can only be done using long-term longitudinal data [17]. Previous studies showed that educational level may be subject to a cohort effect [23,25,49]. Although cohort patterns in educational level may be culturally specific, many Western countries show a similar pattern as the Netherlands regarding educational level across age groups [26]. This may be an indication that our findings are generalizable to most other Western countries were younger people are higher educated than older people. Income might be more dependent on age itself [27], although cohort effects may also exist [49]. Across cohorts, income seems to become more important in relation to health [49]. This might be an explanation for the larger absolute inequalities in physical and mental health by household income in younger compared to older age groups and it might explain part of the U-shape we found for physical and mental health by household income in men. Although a distinction between age and cohort effects was not possible, this study clearly shows that findings on socioeconomic health inequalities are affected by the age of the study population. Third, we could not adjust household income for the number of household members because participants indicated their household income through rather broad pre-specified categories. Although some methods exist to adjust categorical household income for the number of household members, other issues arise. Dividing households into households of 1 or ≥2 persons is one way to correct household income for the number of household members [50]. However, the ordinal scale will be lost. Another method is to use the category middle in a formula that corrects household income for the number of household members. However, no category middles for the extreme categories existed. Therefore, we chose to present crude household income but acknowledge that non-adjustment for the number of household members could affect the findings.

The results of this study may have important implications for researchers and policy makers. Researchers should be aware that the age of their study sample may affect findings on socioeconomic health inequalities. In addition, findings may differ depending on the chosen indicator of SEP and health outcome. We believe that age should be considered equally important as the indicator of SEP and the health outcome when examining socioeconomic health inequalities. Moreover, previous research into socioeconomic health inequalities should be interpreted with caution when age has not been carefully considered as an influential factor on the relationship between SEP and health. Policy makers may want to consider targeting interventions to reduce socioeconomic health inequalities to specific age groups, as the need for reduction of inequalities may differ between the age groups. Reducing absolute health inequalities may be given priority over reducing relative health inequalities as this may be most beneficial for people in lower socioeconomic groups [51]. Especially people between 25 and 50 years old with a primary education or a household income <€1000 per month may need attention as absolute inequalities in physical and mental health are the largest in this age group.

Based on this large-scale cross-sectional study we conclude that absolute and relative socioeconomic health inequalities differ across age groups by indicator of SEP, health outcome and gender. Researchers should be aware of these differences and should critically consider the implications of choosing a specific age group, in addition to the indicator of SEP and health outcome, for research into socioeconomic health inequalities.

## Supporting Information

S1 Tables Tables A-J. (DOCX)

## References

[1] Marmot M (2005) Social determinants of health inequalities. Lancet 365: 1099–1104. 15781105 10.1016/S0140-6736(05)71146-6

[2] Marmot M, Friel S, Bell R, Houweling TA, Taylor S, Commission on Social Determinants of Health (2008) Closing the gap in a generation: health equity through action on the social determinants of health. Lancet 372: 1661–1669. 10.1016/S0140-6736(08)61690-6 18994664

[3] Mackenbach JP, Stronks K (2002) A strategy for tackling health inequalities in the Netherlands. BMJ 325: 1029–1032. 12411368 10.1136/bmj.325.7371.1029 PMC1124499

[4] Addo J, Ayerbe L, Mohan KM, Crichton S, Sheldenkar A, Chen R, et al (2012) Socioeconomic status and stroke: an updated review. Stroke 43: 1186–1191. 10.1161/STROKEAHA.111.639732 22363052

[5] Agardh E, Allebeck P, Hallqvist J, Moradi T, Sidorchuk A (2011) Type 2 diabetes incidence and socio-economic position: a systematic review and meta-analysis. Int J Epidemiol 40: 804–818. 10.1093/ije/dyr029 21335614

[6] Mackenbach JP, Stirbu I, Roskam AJ, Schaap MM, Menvielle G, Leinsalu M, et al (2008) Socioeconomic inequalities in health in 22 European countries. N Engl J Med 358: 2468–2481. 10.1056/NEJMsa0707519 18525043

[7] Chandola T, Ferrie J, Sacker A, Marmot M (2007) Social inequalities in self reported health in early old age: follow-up of prospective cohort study. BMJ 334: 990 17468119 10.1136/bmj.39167.439792.55 PMC1867907

[8] Sacker A, Head J, Gimeno D, Bartley M (2009) Social inequality in physical and mental health comorbidity dynamics. Psychosom Med 71: 763–770. 10.1097/PSY.0b013e3181b1e45e 19622709

[9] Krieger N, Williams DR, Moss NE (1997) Measuring social class in US public health research: concepts, methodologies, and guidelines. Annu Rev Public Health 18: 341–378. 9143723 10.1146/annurev.publhealth.18.1.341

[10] Galobardes B, Shaw M, Lawlor DA, Lynch JW, Davey Smith G (2006) Indicators of socioeconomic position (part 1). J Epidemiol Community Health 60: 7–12. 10.1136/jech.2004.023531 PMC2465546 16361448

[11] Galobardes B, Lynch J, Smith GD (2007) Measuring socioeconomic position in health research. Br Med Bull 81–82:21–37. 17284541 10.1093/bmb/ldm001

[12] Braveman PA, Cubbin C, Egerter S, Chideya S, Marchi KS, Metzler M, et al (2005) Socioeconomic status in health research: one size does not fit all. JAMA 294: 2879–2888. 16352796 10.1001/jama.294.22.2879

[13] Macintyre S, McKay L, Der G, Hiscock R (2003) Socio-economic position and health: what you observe depends on how you measure it. J Public Health Med 25: 288–294. 14747587 10.1093/pubmed/fdg089

[14] Davey Smith G, Hart C, Hole D, MacKinnon P, Gillis C, Watt G, et al (1998) Education and occupational social class: which is the more important indicator of mortality risk? J Epidemiol Community Health 52: 153–160. 9616419 10.1136/jech.52.3.153 PMC1756692

[15] Grundy E, Holt G (2001) The socioeconomic status of older adults: how should we measure it in studies of health inequalities? J Epidemiol Community Health 55: 895–904. 11707484 10.1136/jech.55.12.895 PMC1731799

[16] Geyer S, Hemstrom O, Peter R, Vagero D (2006) Education, income, and occupational class cannot be used interchangeably in social epidemiology. Empirical evidence against a common practice. J Epidemiol Community Health 60: 804–810. 16905727 10.1136/jech.2005.041319 PMC2566032

[17] House JS, Lantz PM, Herd P (2005) Continuity and change in the social stratification of aging and health over the life course: evidence from a nationally representative longitudinal study from 1986 to 2001/2002 (Americans' Changing Lives Study). J Gerontol B Psychol Sci Soc Sci 60 Spec No 2:15–26. 16251586 10.1093/geronb/60.special_issue_2.s15

[18] House JS, Lepkowski JM, Kinney AM, Mero RP, Kessler RC, Herzog AR (1994) The social stratification of aging and health. J Health Soc Behav 35: 213–234. 7983335

[19] Sacker A, Clarke P, Wiggins RD, Bartley M (2005) Social dynamics of health inequalities: a growth curve analysis of aging and self assessed health in the British household panel survey 1991–2001. J Epidemiol Community Health 59: 495–501. 15911646 10.1136/jech.2004.026278 PMC1757050

[20] Herd P (2006) Do Functional Health Inequalities Decrease in Old Age?: Educational Status and Functional Decline Among the 1931–1941 Birth Cohort. Research on Aging 28: 375–92.

[21] Guilley E, Bopp M, Faeh D, Paccaud F (2010) Socioeconomic gradients in mortality in the oldest old: a review. Arch Gerontol Geriatr 51: e37–40. 10.1016/j.archger.2009.12.009 20071040

[22] House JS, Kessler RC, Herzog AR (1990) Age, socioeconomic status, and health. Milbank Q 68: 383–411. 2266924

[23] Lynch SM (2003) Cohort and life-course patterns in the relationship between education and health: a hierarchical approach. Demography 40: 309–331. 12846134 10.1353/dem.2003.0016

[24] Reijneveld SA (2003) Age in epidemiological analysis. J Epidemiol Community Health 57: 397 12775780 10.1136/jech.57.6.397 PMC1732463

[25] Reijneveld SA, Gunning-Schepers LJ (1995) Age, health and the measurement of socio-economic status of individuals. European journal of public health 5: 187–92.

[26] OECD (2012) Education at a Glance 2012: OECD Indicators OECD Publishing.

[27] Kalmijn K, Alessie R (2008) Life course changes in income: An exploration of age- and stage effects in a 15-year panel in the Netherlands Netspar Panel Papers.

[28] European Commission Eurostat (2012) School-to-work transition statistics. Available at: http://ec.europa.eu/eurostat/statistics-explained/index.php/Archive:School-to-work_transition_statistics.

[29] Mackenbach JP, Kunst AE (1997) Measuring the magnitude of socio-economic inequalities in health: an overview of available measures illustrated with two examples from Europe. Soc Sci Med 44: 757–771. 9080560 10.1016/s0277-9536(96)00073-1

[30] Moser K, Frost C, Leon DA (2007) Comparing health inequalities across time and place—rate ratios and rate differences lead to different conclusions: analysis of cross-sectional data from 22 countries 1991–2001. Int J Epidemiol 36: 1285–1291. 17898027 10.1093/ije/dym176

[31] Barros AJ, Victora CG (2013) Measuring coverage in MNCH: determining and interpreting inequalities in coverage of maternal, newborn, and child health interventions. PLoS Med 10: e1001390 10.1371/journal.pmed.1001390 23667332 PMC3646214

[32] Harper S, Lynch J (2005) Methods for measuring cancer disparties: using data relevant to healthy people 2010 cancer-related objectives National Cancer Institute.

[33] Stolk RP, Rosmalen JG, Postma DS, de Boer RA, Navis G, Slaets JP, et al (2008) Universal risk factors for multifactorial diseases: LifeLines: a three-generation population-based study. Eur J Epidemiol 23: 67–74. 18075776 10.1007/s10654-007-9204-4

[34] Scholtens S, Smidt N, Swertz MA, Bakker SJ, Dotinga A, Vonk JM, et al (2014) Cohort Profile: LifeLines, a three-generation cohort study and biobank. Int J Epidemiol 10.1093/ije/dyu229 25502107

[35] Hays RD, Morales LS (2001) The RAND-36 measure of health-related quality of life. Ann Med 33: 350–357. 11491194 10.3109/07853890109002089

[36] VanderZee KI, Sanderman R, Heyink JW, de Haes H (1996) Psychometric qualities of the RAND 36-Item Health Survey 1.0: a multidimensional measure of general health status. Int J Behav Med 3: 104–122. 16250758 10.1207/s15327558ijbm0302_2

[37] Ware JE, Kosinski M, Keller SK (1994) SF-36® Physical and Mental Health Summary Scales: A User's Manual Boston: MA: The Health Institute.

[38] Hays RD, Prince-Embury S, Chen H (1998) RAND-36 health status inventory San Antonio, TX: Psychological Corporation.

[39] Veldman K, Bultmann U, Stewart RE, Ormel J, Verhulst FC, Reijneveld SA (2014) Mental health problems and educational attainment in adolescence: 9-year follow-up of the TRAILS study. PLoS One 9: e101751 10.1371/journal.pone.0101751 25047692 PMC4105412

[40] Porta M (2014) A Dictionary of Epidemiology 6th ed. New York: Oxford University Press.

[41] Regidor E (2004) Measures of health inequalities: part 1. J Epidemiol Community Health 58: 858–861. 15365113 10.1136/jech.2003.015347 PMC1763348

[42] Gini C (1912) Variabilità e mutabilità. Contributo allo Studio delle Distribuzioni e delle Relazioni Statistiche. Bologna: Tipogr. di P. Cuppini.

[43] Hand DJ, Till RJ (2001) A simple generalization of the area under the ROC curve to multiple class classification problems. Machine Learning 45: 171–186.

[44] Ross CE, Wu CL (1996) Education, age, and the cumulative advantage in health. J Health Soc Behav 37: 104–120. 8820314

[45] Westerlund H, Kivimaki M, Singh-Manoux A, Melchior M, Ferrie JE, Pentti J, et al (2009) Self-rated health before and after retirement in France (GAZEL): a cohort study. Lancet 374: 1889–1896. 10.1016/S0140-6736(09)61570-1 19897238

[46] Westerlund H, Vahtera J, Ferrie JE, Singh-Manoux A, Pentti J, Melchior M, et al (2010) Effect of retirement on major chronic conditions and fatigue: French GAZEL occupational cohort study. BMJ 341: c6149 10.1136/bmj.c6149 21098617 PMC2990862

[47] Mein G, Martikainen P, Hemingway H, Stansfeld S, Marmot M (2003) Is retirement good or bad for mental and physical health functioning? Whitehall II longitudinal study of civil servants. J Epidemiol Community Health 57: 46–49. 12490648 10.1136/jech.57.1.46 PMC1732267

[48] Holford TR (1991) Understanding the effects of age, period, and cohort on incidence and mortality rates. Annu Rev Public Health 12: 425–457. 2049144 10.1146/annurev.pu.12.050191.002233

[49] Lynch SM (2006) Explaining life course and cohort variation in the relationship between education and health: the role of income. J Health Soc Behav 47: 324–338. 17240923 10.1177/002214650604700402

[50] Reijneveld SA, Schene AH (1998) Higher prevalence of mental disorders in socioeconomically deprived urban areas in The Netherlands: community or personal disadvantage? J Epidemiol Community Health 52: 2–7. 9604034 10.1136/jech.52.1.2 PMC1756606

[51] Mackenbach JP (2015) Should we aim to reduce relative or absolute inequalities in mortality? Eur J Public Health 25: 185 10.1093/eurpub/cku217 25818489

