# Supplementary material for: Absolute and Relative Socioeconomic Health Inequalities across Age Groups
Source: PLoS One. 2015 Dec 30;10(12):e0145947. doi: 10.1371/journal.pone.0145947 (PMC4696790; doi:10.1371/journal.pone.0145947)
Supplement: S1 Tables — (DOCX) [file pone.0145947.s001.docx]

**Supporting Table A** Physical health by educational level and age group for males

| Age  group | Primary  education | | Lower  secondary education | | Higher  secondary education | | Tertiary  education | |
| --- | --- | --- | --- | --- | --- | --- | --- | --- |
|  | Mean PCS* | n poor/good health** | Mean PCS | n poor/good health | Mean PCS | n poor/good health | Mean PCS | n poor/good health |
| 25-29 | 50.6 | 12/26 | 52.5 | 77/273 | 53.3 | 218/1094 | 54.0 | 130/1028 |
| 30-34 | 49.6 | 18/37 | 51.6 | 151/462 | 52.6 | 311/1194 | 53.9 | 175/1244 |
| 35-39 | 48.3 | 28/33 | 51.1 | 299/734 | 52.3 | 424/1484 | 53.3 | 237/1337 |
| 40-44 | 47.2 | 35/47 | 50.7 | 514/1167 | 52.1 | 573/1993 | 53.4 | 306/1650 |
| 45-49 | 47.6 | 52/64 | 50.3 | 725/1539 | 51.8 | 658/2132 | 52.9 | 383/1789 |
| 50-54 | 47.6 | 35/47 | 50.3 | 435/856 | 51.4 | 398/1081 | 52.5 | 255/1037 |
| 55-59 | 47.4 | 32/25 | 49.5 | 306/551 | 50.4 | 227/463 | 51.6 | 170/517 |
| 60-64 | 48.5 | 56/72 | 49.6 | 359/630 | 50.4 | 208/461 | 51.5 | 187/547 |
| 65-69 | 49.9 | 36/58 | 50.3 | 208/391 | 50.8 | 115/263 | 51.1 | 128/327 |
| 70-74 | 50.3 | 19/37 | 49.9 | 87/165 | 49.6 | 62/113 | 51.2 | 60/148 |
| ≥75 | 49.1 | 24/28 | 47.7 | 64/80 | 48.1 | 35/36 | 49.7 | 29/66 |

* PCS: physical component score

** poor health: PCS<50; good health: PCS≥50

**Supporting Table B** Mental health by educational level and age group for males

| Age  group | Primary  education | | Lower  secondary education | | Higher  secondary education | | Tertiary  education | |
| --- | --- | --- | --- | --- | --- | --- | --- | --- |
|  | Mean MCS* | n poor/good health** | Mean MCS | n poor/good health | Mean MCS | n poor/good health | Mean MCS | n poor/good health |
| 25-29 | 49.7 | 14/24 | 52.0 | 96/254 | 52.6 | 309/1003 | 53.0 | 241/917 |
| 30-34 | 50.3 | 23/32 | 52.3 | 153/460 | 53.0 | 338/1167 | 53.0 | 290/1129 |
| 35-39 | 51.6 | 19/42 | 53.0 | 241/792 | 53.3 | 377/1531 | 53.4 | 313/1261 |
| 40-44 | 48.4 | 33/49 | 53.2 | 384/1297 | 53.3 | 525/2041 | 53.4 | 396/1560 |
| 45-49 | 50.7 | 39/77 | 52.9 | 534/1730 | 53.6 | 544/2246 | 53.7 | 393/1779 |
| 50-54 | 52.7 | 25/57 | 53.5 | 269/1022 | 54.0 | 267/1212 | 53.7 | 249/1043 |
| 55-59 | 53.4 | 15/42 | 54.6 | 155/702 | 54.2 | 128/562 | 54.3 | 109/578 |
| 60-64 | 54.5 | 24/104 | 55.8 | 125/864 | 56.1 | 80/589 | 55.7 | 93/641 |
| 65-69 | 56.0 | 14/80 | 56.6 | 59/540 | 56.7 | 30/348 | 56.9 | 31/424 |
| 70-74 | 56.9 | 7/49 | 57.0 | 19/233 | 57.0 | 17/158 | 56.4 | 21/187 |
| ≥75 | 55.7 | 8/44 | 56.5 | 16/128 | 55.4 | 12/59 | 56.7 | 9/86 |

* MCS: mental component score

** poor health: MCS<50; good health: MCS≥50

**Supporting Table C** Physical health by household income and age group for males

| Age  group | Household income  <€1000 per month | | Household income  €1000 - €2000 per month | | Household income  €2000 - €3000 per month | | Household income  ≥€3000 per month | |
| --- | --- | --- | --- | --- | --- | --- | --- | --- |
|  | Mean PCS* | n poor/good health** | Mean PCS | n poor/good health | Mean PCS | n poor/good health | Mean PCS | n poor/good health |
| 25-29 | 53.5 | 45/201 | 53.2 | 174/821 | 53.6 | 113/693 | 53.8 | 63/480 |
| 30-34 | 52.8 | 26/73 | 52.6 | 170/650 | 52.7 | 232/965 | 53.5 | 160/981 |
| 35-39 | 48.0 | 46/49 | 51.5 | 232/576 | 52.5 | 319/1280 | 52.8 | 276/1314 |
| 40-44 | 47.8 | 50/54 | 51.1 | 293/732 | 52.0 | 500/1606 | 53.0 | 403/1934 |
| 45-49 | 46.9 | 66/61 | 50.1 | 421/862 | 51.6 | 613/1783 | 52.7 | 490/2173 |
| 50-54 | 49.1 | 29/41 | 50.1 | 242/460 | 51.1 | 380/943 | 52.3 | 322/1220 |
| 55-59 | 49.1 | 16/20 | 48.7 | 182/262 | 50.1 | 259/524 | 51.5 | 197/562 |
| 60-64 | 49.3 | 15/24 | 49.0 | 279/401 | 50.8 | 240/568 | 51.5 | 168/517 |
| 65-69 | 51.0 | 4/6 | 49.6 | 152/246 | 51.3 | 172/419 | 51.3 | 94/276 |
| 70-74 | 52.4 | 2/5 | 49.4 | 68/122 | 50.5 | 81/188 | 51.6 | 49/113 |
| ≥75 | 48.8 | 6/5 | 47.5 | 64/69 | 48.8 | 51/76 | 49.2 | 26/44 |

* PCS: physical component score

** poor health: PCS<50; good health: PCS≥50

**Supporting Table D** Mental health by household income and age group for males

| Age  group | Household income  <€1000 per month | | Household income  €1000 - €2000 per month | | Household income  €2000 - €3000 per month | | Household income  ≥€3000 per month | |
| --- | --- | --- | --- | --- | --- | --- | --- | --- |
|  | Mean MCS* | n poor/good health** | Mean MCS | n poor/good health | Mean MCS | n poor/good health | Mean MCS | n poor/good health |
| 25-29 | 49.8 | 88/158 | 51.7 | 283/712 | 53.3 | 163/643 | 54.4 | 80/463 |
| 30-34 | 45.7 | 56/43 | 50.4 | 282/538 | 53.6 | 225/972 | 54.0 | 189/952 |
| 35-39 | 45.8 | 49/46 | 51.5 | 244/564 | 53.5 | 302/1297 | 53.9 | 270/1320 |
| 40-44 | 44.8 | 60/44 | 51.5 | 298/727 | 53.4 | 437/1669 | 54.1 | 392/1945 |
| 45-49 | 45.3 | 72/55 | 51.2 | 391/892 | 53.5 | 473/1923 | 54.4 | 412/2251 |
| 50-54 | 46.9 | 33/37 | 51.9 | 204/498 | 54.0 | 244/1079 | 54.6 | 224/1318 |
| 55-59 | 52.0 | 11/25 | 53.9 | 99/345 | 54.6 | 127/656 | 54.4 | 120/639 |
| 60-64 | 52.0 | 14/25 | 55.0 | 108/572 | 55.7 | 101/707 | 56.4 | 71/614 |
| 65-69 | 54.4 | 2/8 | 56.3 | 41/357 | 56.7 | 48/543 | 57.4 | 16/354 |
| 70-74 | 56.6 | 0/7 | 56.0 | 23/167 | 56.8 | 26/243 | 57.2 | 9/153 |
| ≥75 | 56.9 | 1/10 | 56.5 | 14/119 | 55.9 | 17/110 | 55.9 | 10/60 |

* MCS: mental component score

** poor health: MCS<50; good health: MCS≥50

**Supporting Table E** Physical health by educational level and age group for females

| Age  group | Primary  education | | Lower  secondary education | | Higher  secondary education | | Tertiary  education | |
| --- | --- | --- | --- | --- | --- | --- | --- | --- |
|  | Mean PCS* | n poor/good health** | Mean PCS | n poor/good health | Mean PCS | n poor/good health | Mean PCS | n poor/good health |
| 25-29 | 50.5 | 12/18 | 50.5 | 132/210 | 51.8 | 473/1294 | 53.1 | 374/1731 |
| 30-34 | 49.7 | 18/26 | 50.5 | 206/399 | 51.7 | 547/1462 | 52.9 | 427/1642 |
| 35-39 | 47.9 | 41/35 | 50.1 | 436/819 | 51.8 | 799/2260 | 52.6 | 504/1798 |
| 40-44 | 48.1 | 45/54 | 50.1 | 757/1367 | 51.4 | 1265/3110 | 52.2 | 590/1911 |
| 45-49 | 44.7 | 74/51 | 49.7 | 1131/1940 | 50.7 | 1421/3105 | 51.8 | 670/1971 |
| 50-54 | 47.0 | 56/54 | 49.5 | 764/1208 | 49.9 | 770/1443 | 51.2 | 419/987 |
| 55-59 | 47.5 | 66/65 | 49.1 | 630/960 | 49.7 | 356/607 | 50.7 | 224/494 |
| 60-64 | 47.3 | 94/109 | 48.9 | 741/1012 | 48.9 | 246/359 | 50.2 | 173/351 |
| 65-69 | 48.0 | 71/83 | 49.2 | 427/596 | 49.3 | 121/159 | 49.5 | 96/160 |
| 70-74 | 46.0 | 71/48 | 47.1 | 240/220 | 48.2 | 53/56 | 47.9 | 35/45 |
| ≥75 | 43.1 | 76/33 | 45.8 | 114/70 | 43.7 | 41/12 | 45.3 | 16/9 |

* PCS: physical component score

** poor health: PCS<50; good health: PCS≥50

**Supporting Table F** Mental health by educational level and age group for females

| Age  group | Primary  education | | Lower  secondary education | | Higher  secondary education | | Tertiary  education | |
| --- | --- | --- | --- | --- | --- | --- | --- | --- |
|  | Mean MCS* | n poor/good health** | Mean MCS | n poor/good health | Mean MCS | n poor/good health | Mean MCS | n poor/good health |
| 25-29 | 42.4 | 21/9 | 47.6 | 157/185 | 50.1 | 604/1163 | 51.3 | 608/1497 |
| 30-34 | 48.3 | 19/25 | 48.8 | 247/358 | 50.3 | 677/1332 | 51.0 | 625/1444 |
| 35-39 | 47.2 | 39/37 | 50.0 | 446/809 | 51.2 | 898/2161 | 51.2 | 686/1616 |
| 40-44 | 47.3 | 45/54 | 50.9 | 696/1428 | 51.7 | 1224/3151 | 51.7 | 682/1819 |
| 45-49 | 48.5 | 56/69 | 51.4 | 917/2154 | 52.3 | 1152/3374 | 51.8 | 739/1902 |
| 50-54 | 49.1 | 42/68 | 52.1 | 540/1432 | 52.4 | 553/1660 | 52.2 | 363/1043 |
| 55-59 | 52.6 | 36/95 | 53.0 | 379/1211 | 53.2 | 215/748 | 52.7 | 162/556 |
| 60-64 | 53.0 | 48/155 | 54.0 | 343/1410 | 53.9 | 124/481 | 54.4 | 81/443 |
| 65-69 | 52.5 | 41/113 | 54.0 | 216/807 | 53.6 | 61/219 | 55.0 | 34/222 |
| 70-74 | 53.8 | 30/89 | 54.2 | 91/369 | 52.3 | 33/76 | 55.2 | 11/69 |
| ≥75 | 54.5 | 21/88 | 54.7 | 36/148 | 54.4 | 14/39 | 53.5 | 7/18 |

* MCS: mental component score

** poor health: MCS<50; good health: MCS≥50

**Supporting Table G** Physical health by household income and age group for females

| Age  group | Household income  <€1000 per month | | Household income  €1000 - €2000 per month | | Household income  €2000 - €3000 per month | | Household income  ≥€3000 per month | |
| --- | --- | --- | --- | --- | --- | --- | --- | --- |
|  | Mean PCS* | n poor/good health** | Mean PCS | n poor/good health | Mean PCS | n poor/good health | Mean PCS | n poor/good health |
| 25-29 | 50.8 | 120/246 | 52.1 | 328/946 | 52.6 | 277/977 | 53.1 | 173/797 |
| 30-34 | 50.6 | 73/123 | 51.8 | 301/770 | 52.0 | 388/1167 | 52.7 | 274/1090 |
| 35-39 | 49.1 | 127/162 | 51.5 | 372/906 | 51.6 | 583/1556 | 52.6 | 421/1570 |
| 40-44 | 48.6 | 196/250 | 50.5 | 552/1083 | 51.5 | 752/1961 | 52.2 | 697/2146 |
| 45-49 | 47.4 | 242/253 | 49.9 | 745/1272 | 50.5 | 971/1973 | 51.8 | 781/2410 |
| 50-54 | 47.3 | 139/135 | 49.6 | 453/767 | 50.2 | 549/1033 | 51.1 | 510/1202 |
| 55-59 | 46.5 | 122/95 | 49.2 | 327/490 | 49.5 | 359/583 | 50.8 | 252/573 |
| 60-64 | 47.8 | 105/112 | 48.5 | 391/486 | 49.4 | 317/508 | 50.5 | 183/360 |
| 65-69 | 48.0 | 47/52 | 48.6 | 263/329 | 49.3 | 203/268 | 50.4 | 88/182 |
| 70-74 | 47.6 | 27/24 | 45.2 | 188/131 | 47.8 | 103/100 | 48.3 | 33/46 |
| ≥75 | 44.3 | 37/16 | 44.5 | 119/58 | 45.4 | 33/17 | 42.7 | 20/7 |

* PCS: physical component score

** poor health: PCS<50; good health: PCS≥50

**Supporting Table H** Mental health by household income and age group for females

| Age  group | Household income  <€1000 per month | | Household income  €1000 - €2000 per month | | Household income  €2000 - €3000 per month | | Household income  ≥€3000 per month | |
| --- | --- | --- | --- | --- | --- | --- | --- | --- |
|  | Mean MCS* | n poor/good health** | Mean MCS | n poor/good health | Mean MCS | n poor/good health | Mean MCS | n poor/good health |
| 25-29 | 46.1 | 185/181 | 49.5 | 487/787 | 51.0 | 378/876 | 52.1 | 234/736 |
| 30-34 | 45.5 | 103/93 | 48.8 | 430/641 | 50.3 | 521/1034 | 52.2 | 330/1034 |
| 35-39 | 46.1 | 155/134 | 49.3 | 497/781 | 51.1 | 633/1506 | 52.0 | 504/1487 |
| 40-44 | 47.9 | 200/246 | 50.2 | 595/1040 | 51.4 | 776/1937 | 52.5 | 684/2159 |
| 45-49 | 47.6 | 232/263 | 50.2 | 700/1317 | 52.1 | 811/2133 | 53.0 | 686/2505 |
| 50-54 | 48.4 | 121/153 | 51.3 | 388/832 | 52.5 | 383/1199 | 52.8 | 402/1310 |
| 55-59 | 49.8 | 78/139 | 52.4 | 212/605 | 53.4 | 212/730 | 53.6 | 163/662 |
| 60-64 | 52.2 | 67/150 | 53.2 | 216/661 | 54.4 | 129/696 | 54.7 | 76/467 |
| 65-69 | 52.8 | 23/76 | 53.1 | 144/448 | 54.7 | 85/386 | 55.3 | 39/231 |
| 70-74 | 54.9 | 9/42 | 53.1 | 86/233 | 54.2 | 39/164 | 55.5 | 9/70 |
| ≥75 | 55.2 | 7/46 | 55.1 | 31/146 | 52.9 | 15/35 | 52.6 | 8/19 |

* MCS: mental component score

** poor health: MCS<50; good health: MCS≥50

**Supporting Table I** Gini-coefficients for physical and mental health by educational level and age group

| Age group | Physical health | | Mental health | |
| --- | --- | --- | --- | --- |
|  | Males | Females | Males | Females |
| 25-29 | 0.159 | 0.179 | 0.070 | 0.110 |
| 30-34 | 0.182 | 0.132 | 0.064 | 0.078 |
| 35-39 | 0.181 | 0.130 | 0.046 | 0.056 |
| 40-44 | 0.185 | 0.116 | 0.045 | 0.056 |
| 45-49 | 0.176 | 0.118 | 0.082 | 0.060 |
| 50-54 | 0.163 | 0.091 | 0.052 | 0.039 |
| 55-59 | 0.132 | 0.086 | 0.052 | 0.028 |
| 60-64 | 0.129 | 0.070 | 0.039 | 0.061 |
| 65-69 | 0.081 | 0.043 | 0.125 | 0.093 |
| 70-74 | 0.062 | 0.084 | 0.092 | 0.137 |
| ≥75 | 0.147 | 0.128 | 0.139 | 0.078 |

**Supporting Table J** Gini-coefficients for physical and mental health by household income and age group

| Age group | Physical health | | Mental health | |
| --- | --- | --- | --- | --- |
|  | Males | Females | Males | Females |
| 25-29 | 0.103 | 0.129 | 0.199 | 0.188 |
| 30-34 | 0.112 | 0.115 | 0.243 | 0.181 |
| 35-39 | 0.153 | 0.127 | 0.173 | 0.164 |
| 40-44 | 0.157 | 0.122 | 0.169 | 0.148 |
| 45-49 | 0.180 | 0.159 | 0.204 | 0.175 |
| 50-54 | 0.153 | 0.106 | 0.197 | 0.125 |
| 55-59 | 0.145 | 0.135 | 0.092 | 0.115 |
| 60-64 | 0.165 | 0.110 | 0.134 | 0.189 |
| 65-69 | 0.124 | 0.088 | 0.177 | 0.130 |
| 70-74 | 0.056 | 0.120 | 0.165 | 0.167 |
| ≥75 | 0.107 | 0.046 | 0.082 | 0.185 |
